# Mushrooms: The Good, the Bad, and the Deadly

**DOI:** 10.7759/cureus.102089

**Published:** 2026-01-22

**Authors:** Harikrishna Choudary Ponnam, Ragini Gopagoni, Saketh Parsi, Laxmi Sakamuri, Keyvan Ravakhah

**Affiliations:** 1 Internal Medicine, Summa Health System, Akron, USA; 2 Transplant Medicine, Mayo Clinic, Jacksonville, USA; 3 Hospital Medicine, Ascension Seton Medical Center Austin, Austin, USA; 4 Internal Medicine, Cleveland Clinic Main Campus, Cleveland, USA

**Keywords:** amanita phalloides, amatoxin, liver failure, mushroom poisoning, mycetism

## Abstract

Mushrooms are widely consumed for their culinary and nutritional value; however, some species pose serious toxicological risks. While edible varieties such as *Agaricus bisporus* dominate global and US markets, others, including *Amanita phalloides*, contain potent amatoxins associated with high morbidity and mortality. Mushroom poisoning, or mycetism, occurs across diverse populations and commonly results from accidental ingestion or species misidentification. Diagnosis is often delayed due to difficulties in accurate identification, complicating timely management. This report aims to describe two cases of severe *Amanita* poisoning to illustrate the clinical presentation, diagnostic challenges, and management strategies, including supportive care, antidotal therapy, and consideration for liver transplantation. It also aims to highlight the public health impact of amatoxin-containing mushrooms and underscore the need for early recognition, culturally sensitive education, and effective preventive measures to reduce mushroom-related health risks.

## Introduction

Mycetism is challenging to diagnose due to frequent misidentification, often resulting in delayed recognition and severe hepatotoxicity. Mushrooms are consumed for their umami flavor, largely due to free amino acids and monosodium glutamate-like compounds, with *Agaricus bisporus* being particularly rich in these components [1]. Although over 140,000 mushroom species are estimated worldwide, only about 10% have been identified, and many produce bioactive compounds of pharmaceutical and toxicological importance [2]. Several species are highly toxic, including *Amanita phalloides*, *A. verna*, *A. bisporigera*, *A. virosa*, *A. ocreata*, and *Galerina* species [3].

Mushroom poisoning, or mycetism, affects diverse populations and is often complicated by difficulty in species identification, delaying diagnosis and treatment [4]. In regions with strong foraging traditions, such as the Czech Republic, consumption is substantial [5]. Despite the widespread use of edible mushrooms, toxic look-alike species, particularly within the *Amanita* genus, continue to cause significant morbidity and mortality, emphasizing the need for public health awareness and prevention [6]. We report two cases of severe *Amanita* poisoning and review the clinical spectrum, diagnostic challenges, and current management of mycetism.

## Case presentation

Case 1

An 80-year-old Caucasian man presented to the ED with persistent nausea, vomiting, and abdominal pain for six hours. The initial history was limited due to a language barrier and was subsequently obtained with the assistance of a translator. During evaluation, the patient’s clinical status rapidly deteriorated.

Laboratory studies demonstrated severe hepatocellular injury, with serum transaminase levels exceeding 5,000 U/L (reference range: 9-40 U/L), profound metabolic acidosis with an arterial pH of 6.9, and hypotension. The rapid onset of gastrointestinal symptoms followed by severe hepatic failure, along with the detailed history obtained from the patient’s wife, who reported preparing a homemade soup using mushrooms the patient had collected from a local park, strongly supported mushroom poisoning as the etiology. Other common causes of acute liver failure were excluded on the basis of history, medication review, and initial laboratory testing.

When shown photographs of native mushrooms, she identified the collected species as *Amanita*. A diagnosis of fulminant hepatic failure secondary to mushroom poisoning was made. The patient received aggressive intravenous fluid resuscitation and vasopressor support and was emergently airlifted to a liver transplant center for evaluation. Despite continuous hemodialysis for refractory metabolic acidosis and aggressive supportive care, the patient died several hours later.

Case 2

The patient’s 72-year-old wife was contacted several hours after her husband’s admission, shortly before his transfer for transplant evaluation. Initially, she declined medical evaluation, reporting that she had consumed only a small amount of the mushroom soup and was asymptomatic. After further discussion, she presented to the hospital for evaluation.

Laboratory testing revealed markedly elevated liver transaminases, exceeding 5,000 U/L. Over the ensuing hours, she developed hypotension, coagulopathy, and disseminated intravascular coagulation (DIC). Given her age and comorbidities, she was deemed an unlikely candidate for liver transplantation and was managed conservatively at the presenting facility.

Treatment included IV hydration, vasopressor support, aqueous penicillin G (30 million units daily), and bicarbonate therapy. Her clinical condition gradually improved, with resolution of hemodynamic instability and normalization of liver enzyme levels over the following two weeks. She was discharged home in stable condition.

## Discussion

Mechanism of amatoxin toxicity

Mushrooms, the reproductive structures of Basidiomycetes, have been consumed for centuries for both culinary and medicinal purposes. Certain species exhibit immunomodulatory, anticancer, hepatoprotective, antiviral, and anti-HIV properties, and some have historically been regarded as aphrodisiacs [7].

Amatoxin-containing mushrooms, particularly *Amanita phalloides*, account for the majority of fatal mushroom poisonings. Worldwide, 90% of deaths from mushroom ingestion involve amatoxin-containing species, with *Amanita* species responsible for 90-95% of these fatalities [8]. They contain two main toxin groups: amatoxins (α-, β-, and γ-amanitin), which inhibit RNA polymerase II and cause hepatocellular necrosis, and phallotoxins (phalloidin and phallacidin), which have limited toxicity [9]. Toxin concentrations are highest in the gills and cap, and ingestion of as little as 50 g (two medium-sized mushrooms) can be fatal [10].

Diagnostic challenges

**Figure 1:**
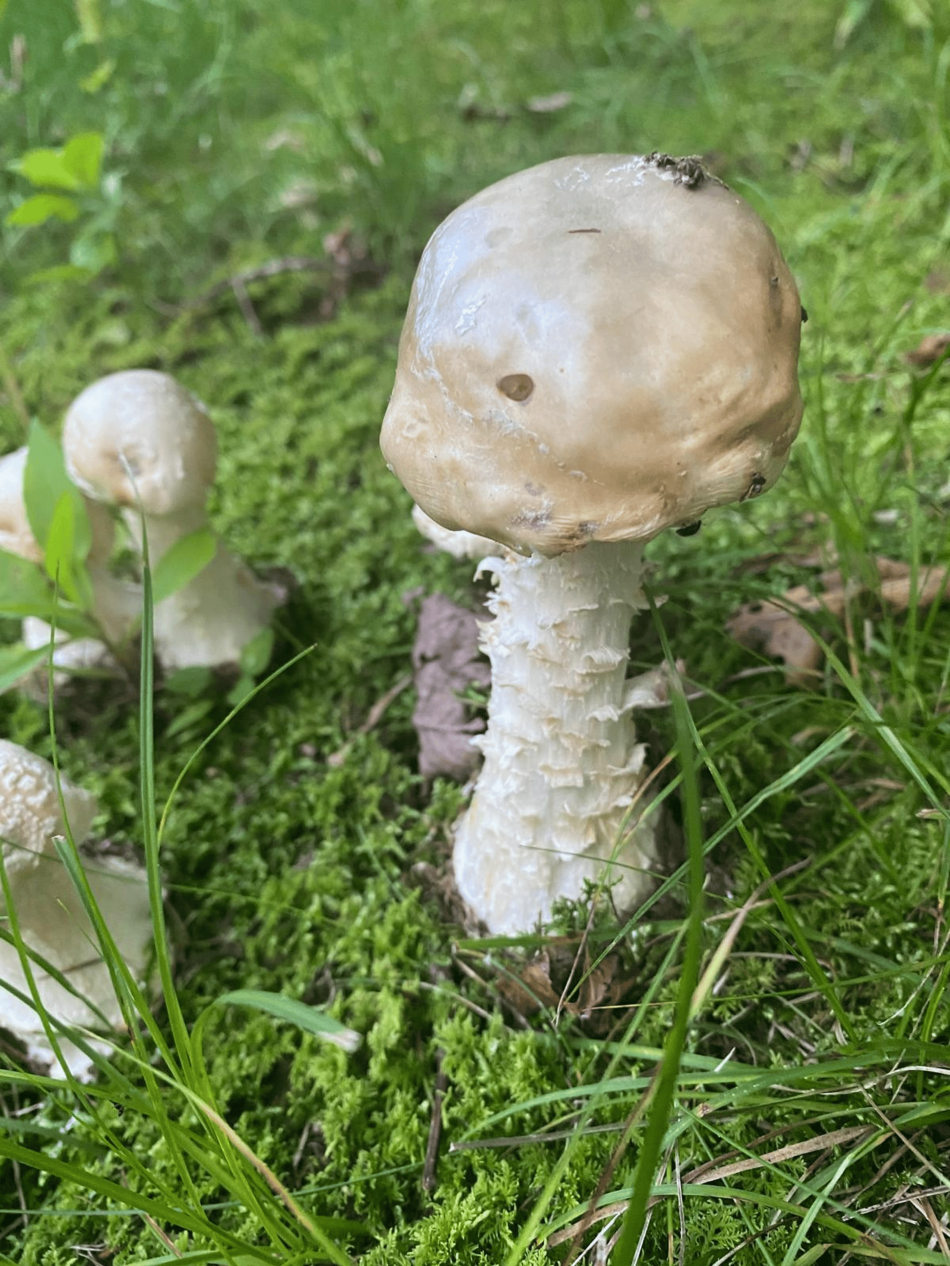
Amanita bisporigera with morphological similarity to edible mushroom species. Original image captured by a co-author; published with permission.

Mushroom poisoning often results from misidentification, as edible species can closely resemble toxic varieties (Figure 1). Of the estimated 5,000 mushroom species found worldwide, only ~25% are named, and ~3% are toxic [11]. Geographic variability and lack of local knowledge increase the risk of accidental ingestion, particularly among immigrants, and the absence of warning signs represents a public health gap [12]. Laboratory detection of amatoxins in urine, blood, tissue, or leftover mushrooms using reverse-phase high-performance liquid chromatography (HPLC) is accurate but not always timely [13].

Management strategies

Early recognition, aggressive supportive care, and targeted therapies are essential. Benzyl penicillin may reduce amatoxin uptake by inhibiting the OATP1B3 transporter [14]. Poor prognostic indicators include diarrhea within 8 hours of ingestion and a prothrombin index <10% after day four, strongly predicting the need for liver transplantation [15].

Public health implications

High-risk populations include children, foragers, individuals with suicidal intent, and immigrants who may confuse local toxic species with familiar edible mushrooms. Because most cases of mushroom poisoning are preventable, increased public awareness of the associated risks is essential to reduce preventable morbidity and mortality [16].

## Conclusions

These cases underscore the critical public health implications of amatoxin-containing mushroom ingestion, particularly from *Amanita phalloides*. By illustrating the clinical severity, diagnostic challenges, and therapeutic urgency of such poisonings, this report emphasizes the need for early recognition, aggressive supportive care, timely antidotal therapy, and consideration for liver transplantation. Moreover, it reinforces the importance of public education, culturally sensitive outreach, and preventive strategies to reduce mushroom-related morbidity and mortality. Documenting these cases provides valuable insight into clinical management and raises awareness of a preventable but potentially fatal health risk.
